# Evaluation of a dry therapeutic urinary diet and concurrent administration of antimicrobials for struvite cystolith dissolution in dogs

**DOI:** 10.1186/s12917-019-1992-8

**Published:** 2019-08-01

**Authors:** Jonathan D. Dear, Jennifer A. Larsen, Michael Bannasch, Sean E. Hulsebosch, Jason W. Gagne, Eric G. Johnson, Jodi L. Westropp

**Affiliations:** 1 Department of Veterinary Medicine and Epidemiology, School of Veterinary Medicine, University of California Davis, Davis, CA 95616 USA; 2 Department of Molecular Biosciences, School of Veterinary Medicine, University of California Davis, Davis, CA 95616 USA; 3 Veterinary Center for Clinical Trials, School of Veterinary Medicine, University of California Davis, Davis, CA 95616 USA; 4 Nestle Purina Pet Care, St. Louis, MO 63103 USA; 5 Department of Surgical and Radiological Sciences, School of Veterinary Medicine, University of California Davis, Davis, CA 95616 USA

**Keywords:** Urolithiasis, Cystolith, Canine, Urinary tract infection, Bladder, Antibiotics

## Abstract

**Background:**

Struvite urolithiasis with bacterial urinary tract infection (UTI) is commonly reported in dogs; few data exist to describe successful dissolution protocols in dogs with naturally occurring disease. We hypothesized that a dry therapeutic urinary diet combined with targeted antimicrobial therapy can effectively dissolve presumptive struvite cystolithiasis in dogs with naturally occurring urease-producing bacterial UTI.

**Results:**

Ten dogs with presumed infection-induced struvite cystolithiasis based on lower urinary tract signs (LUTS), radiodense cystoliths, and urease-producing bacterial UTI were enrolled. At enrollment, antimicrobials and dry therapeutic urinary diet were dispensed. In addition to lack of radiographic resolution of urolithiasis, dogs with persistent clinical signs were considered non-responders. There was no significant difference in pH between responders and non-responders; USG was significantly higher in the responder group. Recheck visits continued until radiographic dissolution or failure was documented. Five of the 10 dogs achieved radiographic dissolution of cystolithiasis within a median of 31 days (range 19–103). In the other 5 dogs, surgical urolith removal was necessary due to persistent LUTS (3 dogs within 2 weeks) or lack of continued dissolution noted radiographically (1 dog with numerous cystoliths failed at day 91; 1 dog failed by day 57 with questionable owner compliance).

**Conclusions:**

Dissolution of urinary tract infection induced struvite cystoliths can be accomplished in some dogs fed this dry therapeutic urinary diet in conjunction with antimicrobial therapy. Case selection could increase the likelihood of successful dissolution; however, if calcium phosphate is present, this could also prevent stone dissolution. If clinical signs persist despite diet and antimicrobials, stone removal is advised.

**Electronic supplementary material:**

The online version of this article (10.1186/s12917-019-1992-8) contains supplementary material, which is available to authorized users.

## Background

Struvite (magnesium ammonium phosphate) containing uroliths are the second most common urolith removed from dogs [1]. The overwhelming majority of canine struvite urolithiasis occurs in females due to host factors which enhance the possibility of bacterial urinary tract infections (UTI). Virtually all canine struvite uroliths are infection-induced, usually by *Staphylococcus pseudintermedius* or, less commonly, by *Proteus mirabilis* or *Klebsiella* spp. These bacteria utilize urease to hydrolyze urea to form ammonia and carbon dioxide, resulting in increased urine pH which liberates ammonium to form magnesium ammonium phosphate crystals. Although struvite uroliths occur most often in the bladder, they can also develop in the kidneys and ureters of dogs [1, 2].

Calculolysis has been reported for feline struvite urolithiasis using dietary therapy and usually occurs within one month but has been reported in as little as 8–14 days [3–5]. Antimicrobial therapy for struvite dissolution in cats is not warranted because struvite uroliths in cats are not associated with UTI as they are in dogs. While dietary dissolution is encouraged for struvite urolithiasis in both species [6] there is a lack of published data regarding efficacy of dietary dissolution in dogs. One study conducted in dogs with struvite urolithiasis and naturally-occurring UTI demonstrated that target urinary pH values that could allow for dissolution of struvite was achieved in all dogs fed either a therapeutic urinary diet or a similar experimental diet for up to 3 months plus antibiotic therapy given for only 1 week; unfortunately, it is unclear if repeated urine cultures and radiographs were performed [7]. Another study showed that a therapeutic urinary diet supplemented with sodium chloride and reduced in protein, phosphorus, and magnesium achieved dissolution of struvite uroliths within an average of 14 weeks (range 2–5 months) in 5/6 Beagle dogs with induced and persistent *S. aureus* UTI without the use of antimicrobials [8]. In this study, outcomes of the UTI were variable with 3/6 clearing infection and 3/6 remaining persistently infected over the study period [8]. It has been suggested that if presumed struvite cystoliths do not begin to decrease in size after approximately 8 weeks of “appropriate therapy” (therapeutic urinary diet plus antimicrobial medication), alternative means of removal should be considered, but some uroliths could take longer to dissolve (up to 7 months has been reported) [9, 10].

The objective of this study was to evaluate the effectiveness of a dry therapeutic urinary diet formulated for struvite dissolution in conjunction with antimicrobial therapy in dogs. We hypothesized that the diet combined with targeted antimicrobial therapy, would lead to effective urolith dissolution in dogs with presumptive struvite cystolithiasis and naturally occurring UTI. The primary outcome was radiographic resolution of cystolithiasis, and secondary outcomes were time to dissolution, clinical signs during dissolution, and adverse effects of this form of therapy.

## Results

Fourteen dogs were screened for eligibility for this clinical trial. Four dogs were excluded; their data were not included for statistical interpretation. One excluded dog had growth of a *Corynebacterium* spp. bacteria on the urine culture, one owner was not compliant with the diet during the first two weeks and was removed at the first recheck visit, one dog had an estimated cystolith that was thought to exceed 85% of the bladder volume and one dog had radiographic evidence of a urolith lodged within the urethra.

Ten dogs completed the trial (Additional file 1: Table S1; 2 mixed breeds, 2 boxers, and 1 each of Great Pyrenees, miniature poodle, Chihuahua, Shih Tzu, Pomeranian, and Newfoundland). The median body weight was 18.8 kg (range 6.1–46.3 kg) and the median age was 5 years (range 2–10.5 years). There were nine spayed females and one castrated male (Additional file 1: Table S1).

**Fig. 1:**
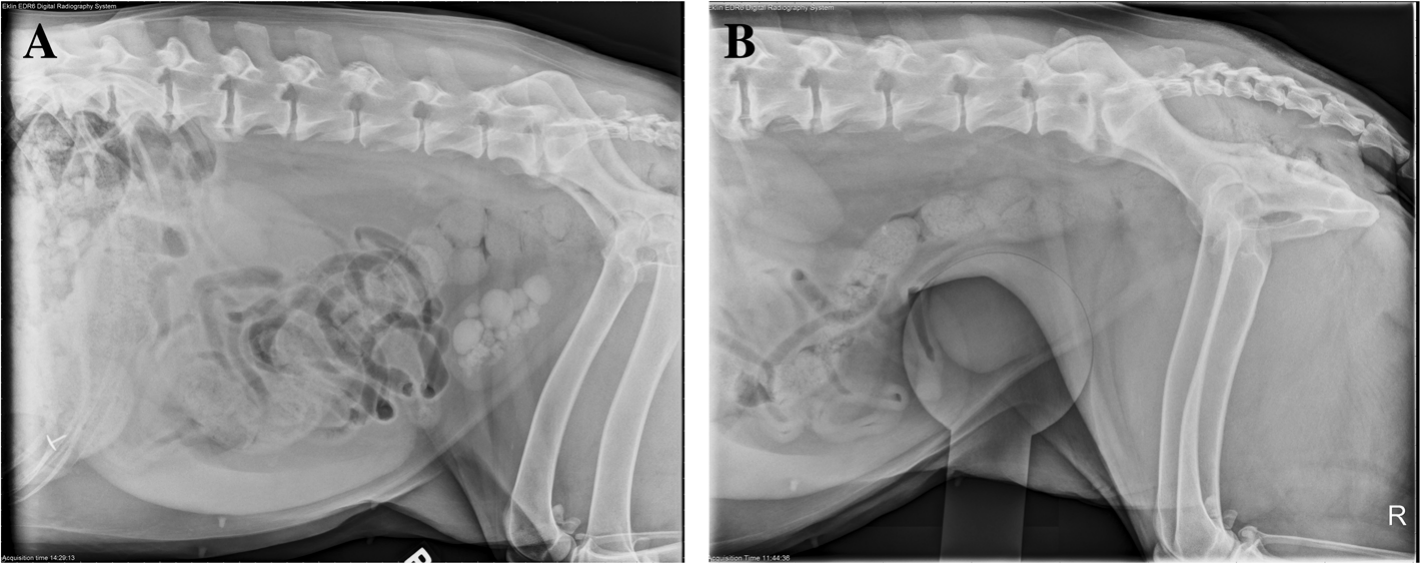
**a**: Lateral projection of a mixed breed dog with presumed struvite urolithiasis (approximately 41 cystoliths) prior to beginning the dry therapeutic urinary diet and amoxicillin. This dog had the largest cystolith in the responder group (2.7 cm). **b**: Lateral projection of the same dog as Fig. 1a with compression of the caudal abdomen by a plexiglass paddle. This dog was radiographed after consuming the diet and amoxicillin for 103 days. No radiographic evidence of cystoliths were noted; ultrasonographic images were also obtained that confirmed these findings

**Fig. 2:**
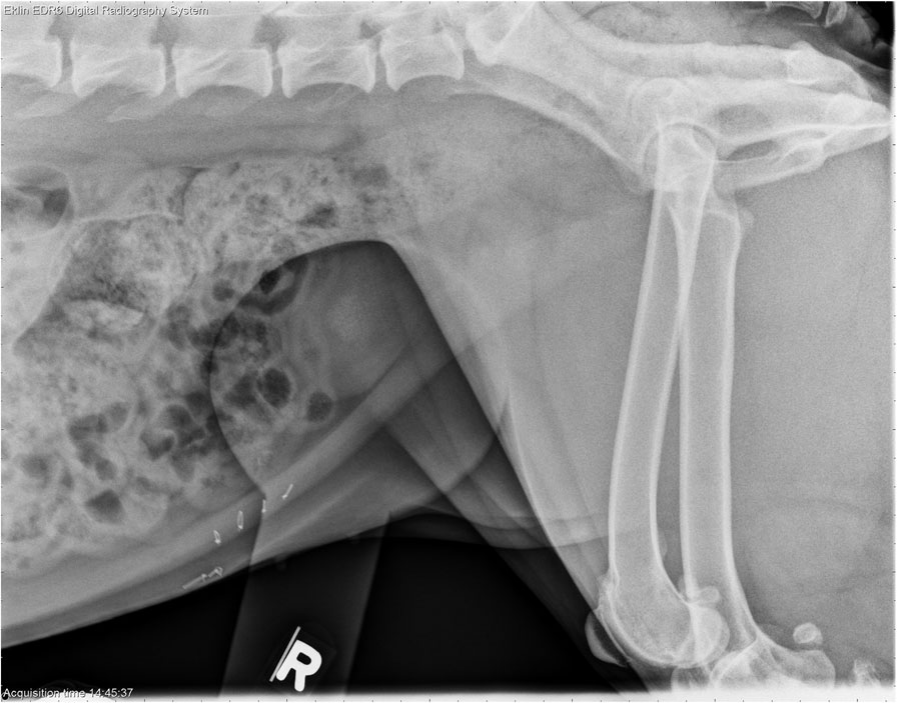
Lateral projection of a Boxer with compression of the caudal abdomen by a plexiglass paddle. Mineral opacities in the urinary bladder are compatible with multiple tiny cystoliths (confirmed via ultrasonography). This dog had the smallest cystolith burden in the responder group, with too numerous to count cystoliths of < 2 mm). No opacities were visible via radiography or by ultrasound on day 19

Five of the 10 dogs (50%, CI 12–88%) were classified as responders based on radiographic resolution of cystolithiasis. The median time to documentation of complete dissolution of cystoliths was 31 days (range 19–103 days). The largest cystolith in the responder group was 2.7 cm (Fig. 1a & b); the number of cystoliths in dogs ranged from 1 to 50. (Fig. 2, Additional file 1: Table S1). Two of the five responders spontaneously voided small cystoliths during the study period.

In the five non-responder dogs, surgical urolith removal was necessary due to persistent LUTS or lack of continued dissolution on serial radiographic assessments, or combination of these two factors. The median time to categorization as a non-responder was 21 days (range 12–94 days). Persistent clinical signs were reported in 3 of these cases at the 2-week visit; all had single uroliths that were surgically removed after this visit (Additional file 1: Table S1). One dog each was prescribed an anti-inflammatory drug or analgesic (carprofen 2 mg/kg PO q12h, tramadol 3.2 mg/kg PO q 8-12 h). Despite this, their clinical signs were not well controlled. The largest cystolith in the non-responder group was approximately 4.5 cm; the number of cystoliths ranged from 1 to 25 (Additional file 1: Table S1).

### Daily urinary diaries

There was not a significant difference in prevalence of LUTS at enrollment or the 2 week visit when dogs were grouped according to outcome. All dogs presented initially for LUTS with stranguria (7; 4 responders and 3 non-responders), pollakiuria (6; 2 responders and 4 non-responders), or hematuria (7; 3 responders and 4 non-responders) or a combination of these signs (4 responders and 4 non-responders). Three dogs were reported to have all three LUTS at enrollment and all were eventually categorized as non-responders. One responder was stranguric at week 2 but this resolved along with dissolution of the cystoliths by week 4. Three non-responders were stranguric at week 2; two of these dogs were considered non-responders at week 4 due to persistent clinical signs, the other dog’s stranguria resolved by week 6 but had persistent cystoliths and was considered a non-responder at week 12.

Two dogs (one responder and one non-responder) were reported to be pollakiuric through the study, though the owners considered this to be ‘normal’ for their dogs, so the dogs remained enrolled in the study. At no point during the study period did they have inappropriate urination in the house, so they was permitted to stay in the study despite mild ongoing clinical signs.

Two responders and all five non-responders had hematuria at enrollment (*P* = 0.16). The hematuria in the two responders (and that of three non-responders) resolved by the 2-week visit.

### Body weight and test diet acceptance

All enrolled dogs maintained body weight within 10% of enrollment weight throughout the study. All of the responders readily ate the diet, with only 1 of the 5 responders losing approximately 6.9% of initial body weight over 12 weeks despite good appetite, and complete consumption of all offered food. Her condition and body weight was closely monitored and the amount offered was increased to slow her rate of weight loss. At enrollment her body condition score was 7/9 and when complete cystolith dissolution was documented, the body condition score was considered ideal at 5/9 [11]. All other responders (4/5) maintained body weight. Of the non-responders, 3/5 did not eat the diet readily; however, their owners reported that they were actively encouraging consumption and diet compliance was presumed in all cases. In fact, only 1 of these 3 dogs lost a small amount of weight (approximately 5.5% of their body weight over 8 weeks while appropriate body condition was maintained throughout the study period). The other 2/3 dogs that did not find the diet palatable but remained weight stable were categorized as non-responders at the 2 week visit due to persistent clinical signs. The last 2 of the non-responders readily consumed all of the diet offered. Despite this, one of those dogs lost 8% of its body weight over 16 weeks; this dog was a 7.3 kg Chihuahua with a body condition score of 9/9.

### Urinalysis and pH

At enrollment, the median urine pH by meter for all dogs was 7.9 (range 6.5–8.2). When grouped according to outcome, there was not a significant difference in urine pH at enrollment or end of study (*P* = 0.52 & *P* = 0.42, respectively). Urine pH at baseline (responders median 7.95, range 6.91–8.11; non-responders median 7.49, range 6.53–8.23) was not associated with response to dietary therapy (*P* = 0.46). Moreover, pH at study completion was significantly lower in both the responders (median 6.73, range 5.45–7.30, *P* = 0.008) and non-responders (median 6.95, range 6.0–7.72, *P* = 0.036) compared to enrollment. The pH change from baseline was not significant between groups (responders median 1.4, range 0.6–2.4, non-responders median 0.8, range 0.3–1.5, *P* = 0.11). At their last visit, 4 dogs had a urine pH less than 6.5, two in each group, there was not a significant difference between groups (*P* = 0.47).

At enrollment, urine specific gravity did not differ significantly between groups. The median USG for the 5 responders was 1.028 (range 1.018–1.037) and for the 5 non-responders was 1.023 (range 1.019–1.039, *P* = 0.72). However, at study completion responder median USG was significantly higher than non-responders (1.034, range 1.031–1.044 vs 1.023, range 1.014–1.034, respectively, *P* = 0.02). Comparing baseline USG to final USG, responders developed significantly higher USG over the study period (*P* = 0.03), while there was no change in non-responders USG (*P* = 0.47).

Nine dogs had bacteriuria and 2 dogs had crystalluria (amorphous in one responder and struvite in two responders) at enrollment. Only 2 dogs had bacteriuria at study completion. One non-responder had persistent cocci seen on urinalysis at the 2 week visit but no growth on aerobic urine culture.

### Microbiology

Every dog enrolled in the trial initially had growth of > 1 × 10^5^ *Staphylococcus pseudintermedius* on aerobic bacterial urine culture. Five dogs (2 responders, 3 non-responders) had growth of 2 strains of *Staphylococcus* spp. These isolates were generally susceptible to most antimicrobials. When possible, antimicrobial selection was made based on susceptibility testing (7 dogs, 4 responders & 3 non-responders). Amoxicillin was determined to be appropriate based on susceptibility testing and was continued in 6 cases (4 responders, 2 non-responders) with a dose range of 18–22 mg/kg by mouth every 12 h while amoxicillin with clavulanic acid was administered based on susceptibility in 2 dogs (both non-responders) at 13–20 mg/kg PO q 12 h. Based on susceptibility patterns, antimicrobial therapy was initiated with enrofloxacin in one non-responder and changed to enrofloxacin in one responder at 8.5–10 mg/kg PO q 24 h.

Only one dog (a non-responder) that originally had growth of *S. pseudintermedius* developed a reinfection at the second visit when a *Mycoplasma* spp. (1 × 10^5^ CFU/mL) was cultured on aerobic bacterial urine culture. No change in antimicrobial therapy was implemented at that visit, but the *Mycoplasma* remained at visit 3 (week 4). Due to this and lack of radiographic dissolution of the cystoliths, doxycycline was administered at 5 mg/kg PO q 12 for 10 days. Subsequent urine cultures remained negative in this dog. However, the cystoliths were static in size and the dog had a cystotomy at week 12. One responder had subclinical bacteriuria (*Enterococcus* spp.) at the final study visit. No antimicrobial treatment was provided.

### Urolith analyses

Urolith analysis was performed on 7 dogs (5 non-responders that underwent cystotomy and 2 responders who spontaneously voided uroliths during the study period). Of the responders, one dog voided 6 uroliths, while the other voided 2 uroliths, all composed of at least 99% struvite (< 1% apatite within the interior). Of the non-responders, quantitative composition analysis of the uroliths that were surgically removed revealed that one dog had single urolith composed of 100% struvite, one dog had single urolith with a struvite core and a minor proportion of apatite in the layers (1–5%), and one dog had single urolith that was primarily struvite with minor (5–10%) urate component in each layer. The 2 other non-responders had multiple uroliths, one with 7 uroliths with struvite cores and 10–50% apatite component in the other layers, and the other with 6 uroliths with calcium oxalate cores and 10–50% struvite and apatite components in the other layers. Due to the nature of the study, analyses could not be performed on all responder dogs; this is similar to previous studies evaluating suspected struvite dissolution in cats [3, 4].

## Discussion

This dry therapeutic urinary diet combined with antimicrobial therapy can effectively dissolve presumed struvite cystoliths in select dogs with urease-producing bacterial UTI. All dogs initially had growth of *S. pseudintermedius* on aerobic bacterial urine culture, which is the most common urease-producing bacterial infection associated with canine struvite cystoliths [12]. All dogs had LUTS, with pollakiuria being the most common complaint by owners at enrollment. The analysis of these pilot data does not support the use of clinical signs at presentation as predictors of outcome. More non-responders had hematuria at enrollment, but the numbers were too small to determine if this is a true association.

The goal of this study was to evaluate this therapeutic urinary diet combined with antimicrobials and we were unable to ascertain if the diet alone was able to relieve LUTS/achieve stone dissolution in these dogs. In infection-induced cystoliths, bacteria grow within a matrix, which is composed of protective exopolysaccharides secreted by the bacteria, and, therefore, could be released during dissolution [13]. Consequently, we opted to treat with antimicrobials throughout the entire dissolution protocol (or until week 16), because this was the conventional approach during the study period [9].

Shorter courses of antimicrobial therapy combined with a calculolytic diet may be efficacious; an induced canine struvite model provides limited evidence for struvite dissolution without antimicrobials [8], however dissolution will likely take longer utilizing this strategy. In one study, the target urinary pH appeared appropriate for struvite dissolution (pH = 5.9–6.1) at day 45 in dogs “showing struvite urolithiasis confirmed by urolith composition assay” with only 7 days of antimicrobial administration during the first week combined with a calculolytic diet. However, radiographic evidence of struvite dissolution was not provided in that manuscript [7]. At this point, no definitive guidelines exist for the timing and duration of antimicrobial therapy in humans or dogs with struvite uroliths [14]. Future studies should consider this to improve antimicrobial stewardship. Recently, recommendations for shorter course therapy are currently being considered for dogs [15].

While there is a published case report demonstrating efficacy of a “noncalculolytic” diet with long- term antibiotic therapy (60 days for presumed struvite dissolution in a dog), the diet administered to the dog was “low fat, protein restricted, and moderately acidifying” compared to its baseline diet which could have been adequate to facilitate dissolution [16]. Further studies could be considered to evaluate struvite dissolution rates utilizing only antimicrobials or dietary therapy alone.

Both of the dogs with single cystoliths that encompassed a large portion of the bladder lumen (3.8 cm and 4.5 cm in dogs that weighed 6.1 and 46.3 kg, respectively), were not only categorized as non-responders, but also had LUTS that did not resolve with diet and antimicrobial therapy. In both dogs, urolith analysis confirmed the composition was primarily struvite, suggesting either the larger urolith size prevented dissolution because the uroliths could not be “adequately bathed in the modified urine” [6] or the large stones present in these dogs were the cause of the persistent LUTS and resulted in recommendations of stone removal via surgery. Though the subjective assessment of urolith burden might have been correct, this criterion may be too ambitious for dissolution and/or the large size prevents amelioration of LUTS due to mechanical irritation of the stone(s) within the bladder. The LUTS were also not able to be controlled in the third non-responder that also had a single urolith (1.7 cm in a 31 kg dog). Likewise, in the fourth non-responder, there was a single urolith present with over 25 cystoliths and “sand” appreciated at enrollment; in addition, several uroliths had a stone composed of 70–80% apatite, which may have acted as a dissolution barrier. Apatite is a common finding in canine struvite uroliths [1], which might hinder success of dissolution therapy, no matter the stone burden. Due to the nature of the study, we do not have complete urolith composition for the 5 responder cases, thus the threshold at which apatite inhibits dissolution remains unknown. The analyses we do have from the 2 responder cases were spontaneously voided. It is our suspicion these stones became small enough to be spontaneously passed during voiding using the calculolytic diet and antimicrobial therapy. All of these stones were primarily struvite and only contained small amounts (< 1%) of apatite.

Urine pH at enrollment or study completion did not differ between responders and non-responders. Despite all dogs having a lower urine pH at study completion, only 4 dogs reached the target urine pH of 6.4. Median reduction in urine pH was higher in responders than non-responders, though this was not found to be significant. These data suggest that pH may not be the only factor influencing outcome, however this pilot study was powered to evaluate dissolution and the small sample size of the study limited the power to completely evaluate this and other secondary outcomes. Furthermore, these were spot urine samples which might not reflect the pH of pooled 24- h urine samples.

Similarly, urine specific gravity did not differ at enrollment between groups but increased during the study period in the responders. Since supersaturation of urine metabolites is a risk factor for cystolith formation, it is often advised to recommend owners to add water to the diet in order to increase intake even though the efficacy of this strategy has not been formally evaluated. The USG noted in these dogs were single point samples and also might not reflect what is occurring in the home environment from day to day. General recommendations for all urolith prevention include “striving to achieve a USG <1.020 for dog” [6]. Data to support this value are lacking and a tailored approach may be needed for each individual dog. Although published data are limited, other studies of canine struvite urolith dissolution protocols reported decreased USG values after switch to calculolytic diets [8]. The increase in USG in responders despite successful dissolution reflects the limited utility of USG in predicting the urine lithogenicity of crystalline compounds in this study. Further, although higher sodium intake in dogs was associated with lower RSS for calcium oxalate urolithiasis, it did not have an effect on USG, but resulted in an approximately 50% increase in daily urine volume and 30% increase in water intake [17]. Data are lacking with regard to the impact of both dietary sodium concentration and water intake on rate and success of struvite dissolution in dogs. Increased water intake may have expedited the dissolution of uroliths in the responders or improved response to medical therapy in the non-responders. However, to avoid confounding the current study with variable water intake, we did not recommend instituting these measures. Moreover, dissolution was still successful in 50% of our study cases despite urine specific gravity > 1.020.

Calorie provision was initially based on owner-reported historic intake, which is inherently imprecise to some extent due to variable types and amounts of treats and owner reliance on volume measurements as well as variable methodology to determine energy density of diets. Although weight loss was not a specific goal for this study, 2 dogs lost 6.9–8% of body weight, and were closely monitored by the investigators. One of these had a 7/9 BCS at enrollment and a 5/9 BCS at successful study completion, and the other dog ended the study (a non-responder) with a BCS that trended from 9/9 to 7/9.

There are several limitations to this pilot study. Some limitations are common to clinical trials, such as accurate confirmation of client compliance not being possible. In addition, although we powered the study to detect the effect of the diet with antimicrobial administration, we might have inadvertently underpowered the study to identify all variables that could influence struvite dissolution (e.g. urine pH, variance in stone size, amount of apatite that could have been present), which was also likely why the confidence interval for response was so wide. The cystolith burden may be a bigger factor in determining response to therapy than previously believed. Case selection may therefore have an impact on expected duration or even the ultimate outcome of medical treatment of canine struvite cystoliths.

## Conclusion

In summary, dissolution of UTI-induced struvite cystoliths can be accomplished in dogs fed this dry urinary therapeutic diet and treated with antimicrobial therapy with appropriate case selection. If the LUTS are successfully controlled, dissolution is progressing, and body weight is stable, dissolution of cystoliths could take up to 4 months. Persistent LUTS and, possibly the apatite component in the uroliths, were reason for dissolution failure.

## Methods

Inclusion criteria included dogs suspected of having struvite cystolithiasis [9]. These were identified as male or female dogs with LUTS, radiodense cystolithiasis and a concurrent urease-producing bacterial UTI with > 1,000 CFU/mL (e.g. *Staphylococcus* spp., *Proteus* spp., and/or *Klebsiella* spp.) confirmed by aerobic bacterial culture of a urine sample collected by cystocentesis.

Exclusion criteria included those dogs where the urolith burden subjectively appeared to involve > 85% of the bladder volume [6] at moderate distention by the radiologist based on established consensus, dogs with *Corynebacterium urealyticum* UTI, dogs consuming diets marketed for struvite dissolution, dogs with documented nephrolithiasis, ureterolithiasis or urethrolithiasis, as well as dogs with bladder masses suggestive of neoplasia noted on abdominal ultrasonography. In addition, dogs treated with antimicrobials or glucocorticoids within 2 weeks of enrollment, or any medication or supplement with the potential to suppress LUTS (e.g. antihistamines, anti-inflammatories, glycosaminoglycans) within 3 days of enrollment were not eligible for enrollment. Finally, dogs with systemic diseases such as diabetes mellitus, hyperadrenocorticism, acute or chronic kidney disease, liver disease, or active pancreatitis were excluded.

All dogs were client-owned, and all owners signed an informed consent. For enrollment screening, dogs were evaluated by one of the study clinicians. A physical examination was performed, and inclusion was considered based on the absence of abnormalities on the CBC and serum biochemical profile. If the urinalysis had evidence suggestive of a urease producing bacterial UTI (e.g. gram-positive cocci), the dog was enrolled, pending final culture results to confirm the pathogen present. Two-view abdominal radiographs were obtained, including radiolucent paddle shots, when necessary, to best image the urinary bladder. Urine was collected by cystocentesis for urinalysis, aerobic bacterial urine culture, and urine pH by meter. Finally, abdominal ultrasonography was performed by a board-certified radiologist or radiology resident under direct supervision of a board-certified radiologist to evaluate for exclusionary criteria.

Owners of enrolled dogs were provided with the dry therapeutic urinary diet^1^ in a bag with nonspecific labeling; manufacturer and brand were masked to the owner. Feeding instructions to maintain historical caloric intake as closely as possible based on provided diet history were provided by a board-certified veterinary nutritionist. Amoxicillin (20 mg/kg PO q12h) was initially prescribed for the UTI when susceptibility results were not available at enrollment. Once urine culture susceptibility results returned, this was changed if resistance to amoxicillin was reported; trimethoprim-sulfa drugs were never used. For some dogs, urine culture and susceptibility was available at enrollment as the sample was obtained by their referring veterinarian. Owners were provided a daily urinary diary to record their dog’s clinical signs throughout the study period (Appendix). If LUTS were not controlled with the diet and antimicrobials, the dog was removed from the study. Pain medications were administered only for the first 3–5 days, if clinically indicated.

Repeat visits were performed at 2, 4, 8, 12, 14, 16, 20, 24, and 28 weeks, or until radiographic dissolution or the dog was characterized as a non-responder as defined below. This time frame was chosen based on the longest time previously reported for dissolution of presumed struvite cystolithiasis in dogs (up to 7 months) [10]. At each visit, a physical examination, abdominal radiographs, and cystocentesis for urine collection were performed. Sedation and administration of enema were allowed if necessary, for adequate radiographic imaging of the urinary bladder. Urine was submitted for urinalysis, urine pH by meter, and aerobic bacterial urine culture. The attending radiologist reported the subjective change in urolith size, if any, from the previous visit based on the 2–3 largest uroliths present. The number of uroliths present in the bladder were also noted. The daily urinary diaries were collected and recorded. Antimicrobials were administered until one week post radiographic resolution (for responders), surgical removal (for non-responders).

In cases of persistent LUTS or if the cystoliths had not completely dissolved by week 28, removal of the urolith was performed. A case was categorized as a non-responder based on 2 criteria: 1) persistent clinical signs of stranguria, pollakiuria or gross hematuria not controlled by week 2 or 2) urolith dissolution was not appreciable after two consecutive visits based on assessment by a radiologist. Any uroliths spontaneously voided or surgically removed were submitted for crystallographic analysis and aerobic urolith culture. A case was classified as a responder when the absence of any uroliths was identified radiographically at any time point during the study; when this occurred, a final abdominal ultrasound was also performed to assess for upper or lower urinary tract mineralization. Urinalysis and aerobic bacterial urine culture via cystocentesis were also performed at the final visit.

## Statistical analysis

This was a single-arm non-controlled prospective phase 2 clinical pilot study based on Simon two-stage design. Using this approach, at least 9 patients with the same histology or molecular target need to be treated with the investigational therapy to test the null hypothesis of insufficient efficacy [18]. As spontaneous resolution of urolithiasis is considered to be rare (estimated less than 5%) a power analysis was performed to detect a response rate of 25% for dogs consuming the dry therapeutic diet and receiving appropriate antimicrobial therapy [19]. Alpha was set at 0.05 and beta was set at 0.8. This determined our recruitment goal of 9 dogs.

Descriptive statistics were used to characterize study dogs. Data were analyzed for normal distribution using the Shapiro-Wilk test and continuous variables were analyzed by paired and unpaired Student’s t-test or Mann-Whitney U test, as appropriate. Repeated measures ANOVA was used to evaluate change in urine pH and USG over time. The Fisher’s exact test was used to evaluate categorical data. Logistic regression was performed to predict response to dietary therapy based on baseline urine pH and presence of clinical signs at baseline and first recheck. *P* < 0.05 were considered significant. All statistical analyses were performed on commercial software.^2^

### Additional file

Additional file 1: **Table S1.** Table of individual patient data including signalment, initial cystolith burden, outcome and stone analysis (when available). Clinical characteristics of enrolled dogs in a trial of a dry therapeutic diet and antimicrobial administration to dissolve presumed struvite cystolithiasis. (DOCX 17 kb)

## Data Availability

The datasets used and/or analyzed during the current study are available from the corresponding author on reasonable request.

## Appendix

**Table 1:** Daily diary provided to owners. Example of multiday diaries provided to dogs’ owners in order to record dairy occurrence of LUTS

Study Day	Date	
1. Does your dog void small amounts of urine frequently?	Yes	No
2. Do you notice blood in your dog’s urine?	Yes	No
3. Do you notice your dog straining to urinate?	Yes	No

## Abbreviations

BCS: Body condition score
LUTS: Lower urinary tract signs
RSS: Relative supersaturation
USG: Urine specific gravity
UTI: Urinary tract infection

## Footnotes

1 Purina® Pro Plan® Veterinary Diets UR® Urinary Ox/St™ Canine Formula dry

2 SAS v. 9.4 (Cary, NC)
