# Supplementary material for: Evaluation of a dry therapeutic urinary diet and concurrent administration of antimicrobials for struvite cystolith dissolution in dogs
Source: BMC Vet Res. 2019 Aug 1;15:273. doi: 10.1186/s12917-019-1992-8 (PMC6676539; doi:10.1186/s12917-019-1992-8)
Supplement: Supplementary file 1 — Table S1. Table of individual patient data including signalment, initial cystolith burden, outcome and stone analysis (when available). Clinical characteristics of enrolled dogs in a trial of a dry therapeutic diet and antimicrobial administration to dissolve presumed struvite cystolithiasis. (DOCX 17 kb) [file 12917_2019_1992_MOESM1_ESM.docx]

Table S1 Clinical characteristics of enrolled dogs in a trial of a dry therapeutic diet and antimicrobial administration to dissolve presumed struvite cystolithiasis.

| Signalment | Number of calculi | Size of calculi | Outcome | Stone analysis |
| --- | --- | --- | --- | --- |
| 2 Y FS Great Pyrenees | 24 calculi | 2mm-1.3cm | Resolved at week 4 | N/A |
| 12 Y FS Labrador X | 41 calculi | 2mm-2.7cm | Resolved at week 13. | 99-100% struvite cystoliths voided at visit 0 |
| 5 Y FS boxer | TNTC sand and small calculi | All <2mm | Resolved at week 2. | N/A |
| 4 Y MC miniature poodle | Single calculus | 9mm | Resolved at week 8. | N/A |
| 10 Y FS Shih Tzu | 50 calculi | 2-9mm | Resolved at week 6. | 99-100% struvite cystoliths voided at visit 0 |
| 3 Y F terrier X | 20 calculi, moderate sand | 2-9mm | Lower burden but largest remains 8mm at week 8. Cystotomy. | Larger cystoliths core 90% struvite, outer layer 20-40% apatite |
| 6 Y FS Chihuahua | 25 calculi, moderate sand | 2mm-1.4cm | Unchanged burden at week 12. Cystotomy. | Numerous 1-12mm cystoliths removed via surgery. Core 90% CaOx, outer layer 90% struvite |
| 3 Y FS boxer | Single calculus | 1.7cm | Unchanged size at week 2. Cystotomy due to persistent signs. | 90% struvite, 10% urate |
| 15 Y FS Pomeranian | Single calculus | 3.8cm | Unchanged size at week 2. Cystotomy due to persistent signs. | 95% struvite, 5% apatite |
| 5 Y FS Newfoundland | Single calculus | 4.5cm | Unchanged size at week 2. Cystotomy due to persistent signs. | 100% struvite |
